# A synthesis of mercury research in the Southern Hemisphere, part 2: Anthropogenic perturbations

**DOI:** 10.1007/s13280-023-01840-5

**Published:** 2023-03-23

**Authors:** Jenny A. Fisher, Larissa Schneider, Anne-Hélène Fostier, Saul Guerrero, Jean Remy Davée Guimarães, Casper Labuschagne, Joy J. Leaner, Lynwill G. Martin, Robert P. Mason, Vernon Somerset, Chavon Walters

**Affiliations:** 1 Centre for Atmospheric Chemistry, School of Earth, Atmospheric and Life Sciences, University of Wollongong, Northfields Avenue, Wollongong, NSW 2522 Australia; 2 College of Asia and the Pacific, Australian National University, Coombs Bld 9 Fellows Rd, Acton, Canberra, ACT 2601 Australia; 3 Instituto de Química/Unicamp, Rua Josué de Castro, s/n – Cidade Universitária, Campinas, SP 13083-970 Brazil; 4 Lab. de Traçadores, Instituto de Biofísica, Centro de Ciências da Saúde, Bloco G, Av. Carlos Chagas Filho 373, Ilha do Fundão, Rio de Janeiro, CEP 21941-902 Brazil; 5 South African Weather Service c/o CSIR Environmentek, 11 Jan Cilliers Street, Stellenbosch, 7599 South Africa; 6 Department of Environmental Affairs and Development Planning, Western Cape Government, Property Building, 1 Dorp Street, Cape Town, 8001 Western Cape South Africa; 7 Atmospheric Chemistry Research Group, Chemical Resource Beneficiation, North-West University, Potchefstroom, 2520 South Africa; 8 Department of Marine Sciences, University of Connecticut, 1080 Shennecossett Road, Groton, CT 06340 USA; 9 Department of Chemistry, CPUT, CPUT Bellville Campus, Bellville, 7535 Western Cape South Africa; 10 Council for Scientific and Industrial Research, 11 Jan Cilliers Street, Stellenbosch, 7599 South Africa

**Keywords:** ASGM, Deforestation, Legacy mercury, Mercury emissions, Methylmercury, Southern Hemisphere

## Abstract

Environmental mercury (Hg) contamination is a global concern requiring action at national scales. Scientific understanding and regulatory policies are underpinned by global extrapolation of Northern Hemisphere Hg data, despite historical, political, and socioeconomic differences between the hemispheres that impact Hg sources and sinks. In this paper, we explore the primary anthropogenic perturbations to Hg emission and mobilization processes that differ between hemispheres and synthesize current understanding of the implications for Hg cycling. In the Southern Hemisphere (SH), lower historical production of Hg and other metals implies lower present-day legacy emissions, but the extent of the difference remains uncertain. More use of fire and higher deforestation rates drive re-mobilization of terrestrial Hg, while also removing vegetation that would otherwise provide a sink for atmospheric Hg. Prevalent Hg use in artisanal and small-scale gold mining is a dominant source of Hg inputs to the environment in tropical regions. Meanwhile, coal-fired power stations continue to be a significant Hg emission source and industrial production of non-ferrous metals is a large and growing contributor. Major uncertainties remain, hindering scientific understanding and effective policy formulation, and we argue for an urgent need to prioritize research activities in under-sampled regions of the SH.

**Supplementary Information:**

The online version contains supplementary material available at 10.1007/s13280-023-01840-5.

## Introduction

In recent years, improved understanding of the natural biogeochemical cycling of mercury (Hg) has been coupled with advances in identifying and predicting the impacts of human perturbations on the Hg cycle (Obrist et al. 2018). Collectively, these advances have increasingly been used as the scientific basis for Hg regulation and policies worldwide. However, the majority of Hg data that underlies current scientific understanding comes from the Northern Hemisphere (NH) and in many cases is not representative of the conditions (historical, environmental, and socioeconomic) of the Southern Hemisphere (SH). In this paper, we identify and describe the four primary human-influenced differences between the hemispheres that influence present-day Hg emission and mobilization processes. As the tropical countries span both hemispheres, they are included in the synthesis in conjunction with the SH when appropriate. A companion paper (Schneider et al. 2023) describes the hemispheric differences in natural Hg processes.

Historical, political, cultural and socioeconomic differences between NH and SH countries have led to different anthropogenic perturbations to natural Hg cycling between the hemispheres. In the SH, economic inequality, lower population numbers, a history of Hg production and silver refining, higher dependency on fire for energy and land clearing, deforestation and agricultural expansion, less stringent regulations, and the use of Hg in artisanal and small-scale gold mining (ASGM) collectively combine to create differences relative to the NH in Hg biogeochemical cycling. These differences influence not only present-day Hg sources but also emissions of legacy Hg from historical activities as well as Hg cycling between reservoirs. Given that the timescales for interhemispheric air exchange (~ 1–1.5 years) (Patra et al. 2011; Yang et al. 2019) are longer than the atmospheric lifetime of elemental Hg (~ 3–6 months) (Horowitz et al. 2017; Saiz-Lopez et al. 2020; Shah et al. 2021; Zhang and Zhang 2022), and that oxidized forms of emitted Hg are removed much more rapidly, these perturbations (especially those impacting Hg emissions) will lead to hemispheric differences in deposition signatures (Corbitt et al. 2011; Driscoll et al. 2013).

The relationship between Hg emissions, transport and deposition is central to understanding the causal chain from policy to impacts, with important implications for governments formulating and evaluating Hg policy options under the Minamata Convention (Selin et al. 2018). However, in the absence of specific and comprehensive Hg data from the SH, findings based mostly or exclusively on NH data, especially from temperate regions, are often assumed to be broadly applicable and are extrapolated to the SH. Such a perspective ignores the unique features of the SH that influence Hg cycling and is particularly concerning in the tropics, where a paucity of Hg data intersects with a large fraction of global Hg emissions and a need for wide-ranging Hg policy measures specifically suited to the region (Selin et al. 2018).

**Fig. 1:**
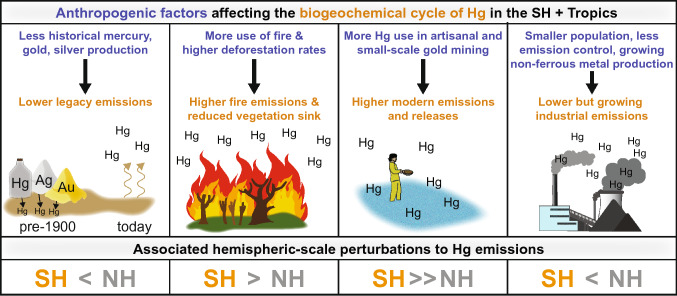
Conceptual overview of the key hemispheric differences described in this paper, showing how anthropogenic factors differ in the SH and tropics relative to the NH (blue) and the associated impact on Hg biogeochemical cycling (orange). The bottom row indicates how the associated hemispheric-scale perturbation to Hg emission compares between hemispheres

The purpose of this synthesis paper is to review current understanding of anthropogenic perturbations to the Hg cycle through an SH- and tropics-focused lens. Previous Hg syntheses highlighted the importance of location-specific knowledge for linking Minamata Convention policies to their impacts and effectiveness (Selin et al. 2018), with the SH and tropics specifically identified as the regions where data and scientific understanding are most lacking (Obrist et al. 2018). Together with the companion paper on natural processes (Schneider et al. 2023), this synthesis effort summarizes what is currently known about mercury sources and cycling in the SH and where the most critical knowledge gaps remain. Here, we identify and describe four human-driven differences between the hemispheres that have implications for SH and tropical Hg cycling: the historical legacy of Hg use, the prevalence of fire and deforestation, ASGM, and Hg emissions associated with industrial sources. Figure 1 shows the four human-driven perturbations described in this paper, spanning historical and modern timescales with influence on Hg cycling across atmospheric, terrestrial and aquatic reservoirs. We focus on perturbations of most relevance to the SH mid-latitude and tropical regions, extending to the NH tropics when relevant (collectively referred to in this context as the SH + T). In the sections that follow, we describe each perturbation in the context of differences between the hemispheres. In the process, we review the existing literature to synthesize the current state of knowledge on the response of SH Hg cycling to anthropogenic perturbations. Finally, we highlight key knowledge gaps that limit scientific understanding and the ability to formulate effective Hg policy in the SH.

## Legacy mercury

The persistent nature of Hg in the environment means that legacy Hg continues to contribute to the current biogeochemical cycling of Hg, with re-emission from both ocean and terrestrial reservoirs. Here, we define legacy Hg as anthropogenic Hg emissions and releases that took place in the past, either at the time Hg was produced or employed in a manufacturing process or gradually (in the intervening years to the present) from historic end- or by-products containing Hg. The magnitude of legacy Hg emission from different historical periods has a direct bearing on the efficiency of Hg control policies adopted under the Minamata Convention, since the greater its contribution to the present-day Hg cycle, the greater the need for more stringent measures to reduce current levels of anthropogenic Hg in the environment (Amos et al. 2013; Horowitz et al. 2014). In the absence of pre-1900 on-site measurements of historic anthropogenic Hg, our current understanding of legacy Hg emissions and releases from this period is based on bottom-up estimates (e.g., Streets et al. 2019) combined with measurements of Hg deposition flux from natural archives such as lake sediment and peat bogs (e.g., Engstrom et al. 2014; Cooke et al. 2020). Both approaches require integrating information from historical archives with environmental research.

The current estimates of pre-1900 historical Hg emission and release have been based on an assumption that silver, Hg and gold production were the main cause of a significant increase of Hg in the atmosphere, although the extent thereof remains the subject of debate (Engstrom et al. 2014; Outridge et al. 2018; AMAP/UNEP 2019; Streets et al. 2019). The published literature on historical analysis of pre-1900 Hg use show that the majority of Hg used for historical silver refining was immediately sequestered in solid calomel as a by-product of the refining reaction (Johnson and Whittle 1999), but the timeline of any subsequent Hg releases from this significant pool of potential legacy Hg is unknown (Streets et al. 2019; Loria et al. 2022). In addition, not all the silver and gold produced before 1900 utilized Hg (Egleston 1887, 1890). The breakdown of global silver and gold production data according to which Hg recipe (with or without iron) and process (*patio*, *cazo*, pan) was used, whether smelting with lead was performed, or whether simple separation by gravity was used (for placer gold), is still pending. Until these data become available, any projection of legacy Hg emissions and releases based solely on total silver or gold production without accounting for the Hg recipe and process should be treated with caution, limiting our ability to make hemispheric comparisons.

Beyond silver and gold production, there is an imbalance between sources and uses of anthropogenic Hg across the two hemispheres, with more historical Hg use in the NH. Production of Hg, a critical source of Hg emissions, has always been more important in the NH, where 86% of all Hg produced from 1501 to 1900 was sourced (Hylander and Meili 2003). In addition, there was also more historical industrial activity and associated consumption of Hg in the NH than the SH, noting that industrial-scale Hg consumption included both industrialized and non-industrialized countries in the nineteenth century. Total silver and gold production up to 1900 were significant in the NH (New Spain/Mexico, USA), with Hg used in some cases (Guerrero 2016). Historical research has identified China as a major importer of Hg from mines in California (USA) after 1850, with the Hg used for the manufacture of vermilion (St. Clair 1994). As these examples show, it is now possible to convert a wider sample of archival evidence (beyond the history of silver and gold production) into a quantitative assessment of the hemispheric differences in historical Hg use. More detailed country-level historical trade, production and end-market data are needed to provide critical context for sediment core analysis and more accurate apportionment of legacy Hg sources between the two hemispheres during this period.

Turning to deposition flux measurements, there are few studies that have specifically compared the contribution of pre-1900 Hg emissions between the NH and SH. Most of these studies were conducted using lake sediments and peat bogs to reconstruct spatial and temporal patterns in past atmospheric Hg deposition. However, interpretation of Hg deposition in these archives requires caution, as accurate interpretation of these archives necessitates consideration of the effects of the local physical and ecological dynamics on the Hg cycle (Cooke et al. 2020). For instance, peat decomposition, lead (^210^Pb) and Hg mobility, and Hg export from watershed to lake collectively make the interpretation of cores as a proxy for global legacy Hg emission a challenging task. As a result of these challenges, there remain discrepancies in the literature regarding the hemispheric distribution of legacy Hg deposition.

Most studies on preindustrial Hg deposition in lake sediment archives indicate that Hg emissions from the preindustrial period (prior to 1850) were deposited locally near urban and industrial centers of Hg use and release (Cook et al. 2022). A study of Hg fluxes in lakes in Nova Scotia (NH) and New Zealand (SH) did not show evidence of a global-scale impact from historic silver or gold production at either location, up to at least the 1850s (Lamborg et al. 2002). The change in Hg deposition at the NH site was up to twofold larger than at the SH site by the year 2000 (Lamborg et al. 2002).

More broadly, a consistent threefold to fivefold increase in Hg deposition from the pre-anthropogenic period (< 1850 AD) to the late twentieth century has been reported for both hemispheres (Lindberg et al. 2007; Engstrom et al. 2014), suggesting similar historical amounts of Hg release in both hemispheres. This result is potentially at odds with the larger Hg production and use in the NH seen in the historical records and highlights the need for a better understanding of the relationships between Hg production, use, and emissions by different industrial activities. In contrast, a recent review of all-time enrichment, as recorded in lake sediment and peat records, proposed a fourfold enrichment in the SH compared to a 16-fold enrichment in the NH (Li et al. 2020). This equates to fourfold larger Hg deposition in the NH than in the SH, which is the highest hemispheric difference reported in the literature. The authors attributed this difference to lower post-1800 anthropogenic Hg emissions in the SH and higher natural atmospheric Hg concentrations, contributing to a higher background Hg accumulation rate in the SH. Some caveats have been raised about this interpretation of a large hemispherical difference, including the reliability of peat cores as faithful archives of past Hg deposition and biases from confounding local versus global Hg pollution in lake sediments (Cooke et al. 2022).

A further challenge for interpreting hemispheric-scale legacy Hg emission inferred from natural archives is the bias caused by the different number of studies, which is much greater in the NH, where they also encompass a larger geographical extent than in the SH (Li et al. 2020). With far fewer records, trends in the SH are currently not as robust as for the NH. For instance, the fewer study sites in the SH are mostly located in coastal areas, skewing the results towards locations with significant oceanic emissions (Schneider et al. 2023). In addition, the assumed magnitude of preindustrial Hg emission is based largely on inventories of historical Hg use and estimated emission factors (Engstrom et al. 2014), which, given the uncertainties in these historical inventories described above, may lead to further uncertainties in the rate of change over time. Until we have a more robust historical understanding of Hg use and release and a more comprehensive SH sediment core dataset, reconciling natural archives with one another and with the historical data will remain a challenge.

## Fire and deforestation

Fires are major sources of global atmospheric Hg, releasing Hg sequestered in vegetation and soil by volatilization and thermal desorption (Outridge et al. 2018). Elevated atmospheric Hg concentrations have been observed in fire smoke plumes throughout the SH, in South America (Artaxo et al. 2000; Michelazzo and Fostier 2006; Ebinghaus et al. 2007), Africa (Brunke et al. 2001; Angot et al. 2014), and Australia (Desservettaz et al. 2017; Howard et al. 2017, 2019). Fires also release Hg from soil to aquatic systems by removing vegetation cover, favoring soil lixiviation (leaching) and erosion and Hg transport to water, where methylmercury (MeHg) can be produced and become available for bioaccumulation in aquatic organisms (Amirbahman et al. 2004; Dittman et al. 2010).

Fire patterns differ significantly between the hemispheres, with the majority of both annual area burned and fire carbon emissions in the SH + T (van der Werf et al. 2017). The prevalence of savanna ecosystems in the SH + T leads to more frequent fires (shorter return intervals) on average in the SH + T than in the extratropical NH (where the bulk of burnt area and emissions come from boreal forest fires). Natural fires are augmented by anthropogenic fires, most notably from deforestation, which is most prevalent in Africa and South America (FAO 2020). Hemispheric differences in fire are partly economic and cultural in origin. In developing economies (more prevalent in the SH), fires are used for a variety of activities that have largely been industrialized in wealthier countries, including clearing forest and bushland for agriculture; controlling pests, insects, and weeds; preventing litter accumulation to preserve pasturelands; mobilizing nutrients; producing charcoal for industrial and domestic use; providing energy for residential cooking and heating; and managing waste (Crutzen and Andreae 1990; Zulu and Richardson 2013; Wiedinmyer et al. 2014). Furthermore, these countries typically have limited funds to invest in wildland fire management programs (Brady et al. 2007). Deforestation (permanent conversion from forest to non-forest land) is a primary cause of anthropogenic fire and associated Hg mobilization in the SH and is discussed further below.

### Hg emissions to the atmosphere from fire

Distinguishing between natural and anthropogenic Hg fire emissions is complex given the legacy Hg accumulated in terrestrial ecosystems and the anthropogenic impacts on fire occurrence and intensity. We therefore discuss both together here. Model estimates of Hg emissions to the atmosphere from biomass burning (both anthropogenic and wildfires) suggest that tropical fires are responsible for the majority of global fire Hg emissions (Friedli et al. 2009; De Simone et al. 2015; Kumar et al. 2018; Shi et al. 2019). Tropical biomass burning Hg emissions are dominated by forest fires (61%) followed by fires in woody savanna/shrubland (30%) and savanna/grassland (7%) (Shi et al. 2019).

**Fig. 2:**
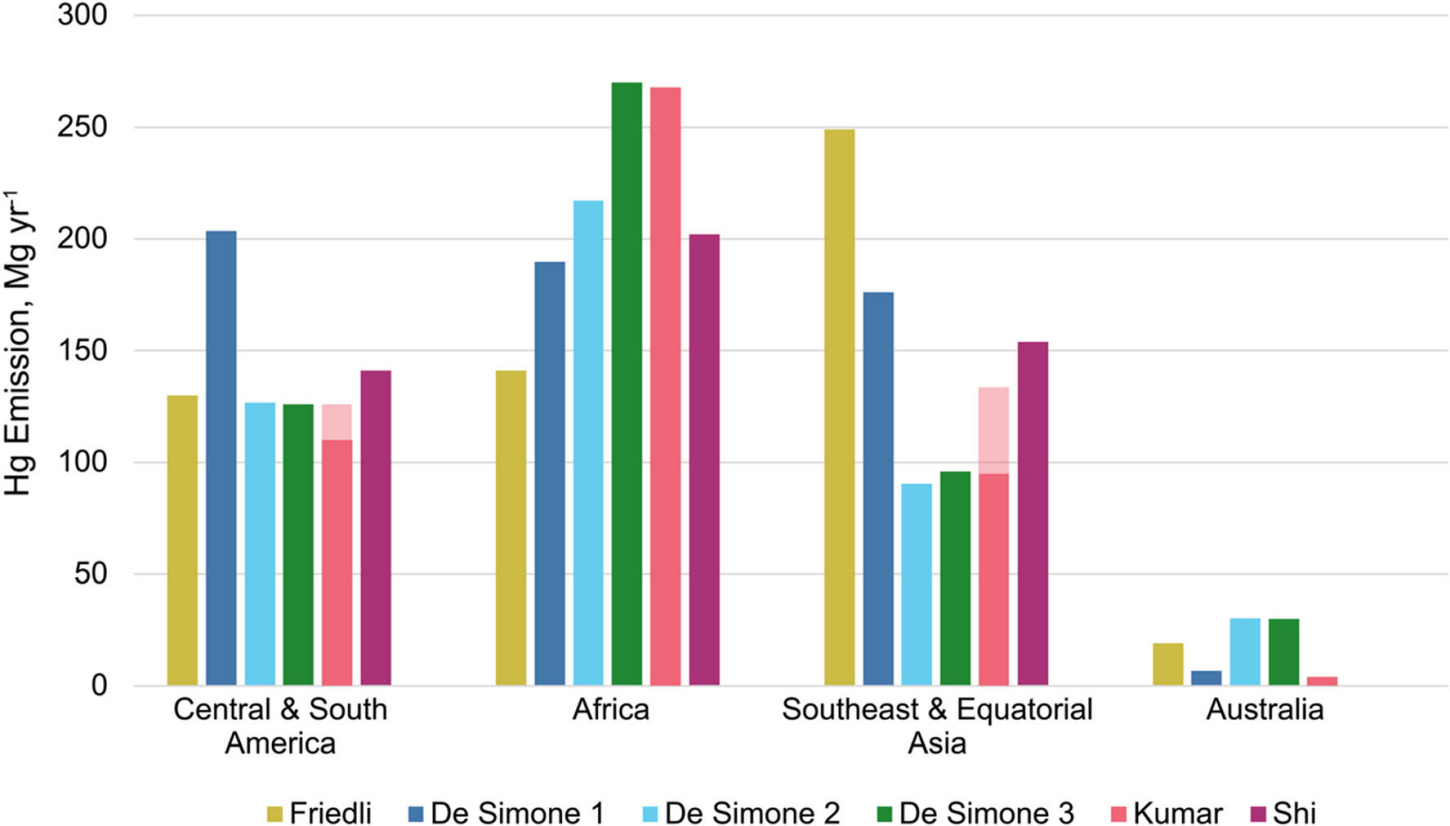
Estimates of Hg emissions from fires in the SH + T. Estimates are based on data published in Friedli et al. (2009), De Simone et al. (2015), Kumar et al. (2018), and Shi et al. (2019). De Simone 1, 2, and 3 refer to FINN, GFAS, and GFED, respectively, from De Simone et al. (2015). Regional data from Friedli et al. (2009) and De Simone et al. (2015) have been summed as follows: Central & South America = CEAM + NHSA + SHSA; Africa = NHAF + SHAF; Southeast & Equatorial Asia = SEAS + EQAS; Australia = AUST (with shorthand names defined in those papers). Kumar et al. (2018) used somewhat different regional definitions than those used here. For this plot, we used the results from De Simone et al. (2015) to calculate the fractional contribution of each sub-region (e.g., SEAS) to each of the Kumar et al. (2018) regions (e.g., EURAS) to re-calculate the estimated regional totals. The variation in the fractional contributions results in low- and high-end estimates shown in the dark and light colors, respectively, for the Kumar bars (pink). Note that Shi et al. (2019) did not provide an estimate for Australia

Figure 2 shows published estimates of Hg emissions from fires in the SH + T. These estimates use different methodologies and datasets for estimating fire activity (e.g., fire counts, burned area, fire radiative power) and resultant Hg emissions (see Table S1 in the Supplementary Information). The largest Hg emissions from fires are generally estimated to come from Africa (including both NH and SH Africa), with somewhat smaller but still sizable contributions from tropical Asia and South America. Observed increasing trends in atmospheric Hg in South Africa since 2007 have been attributed to emissions from biomass burning in South America and southern Africa (Martin et al. 2017). Tropical Australian savanna fires are important Hg sources locally but less important globally (Howard et al. 2019; Fisher and Nelson 2020) due to lower fuel consumption than in other SH ecosystems (van der Werf et al. 2017), combined with low Hg emission factors from Australian savanna vegetation (Desservettaz et al. 2017).

Despite their outsize contribution to global emissions, there is significant uncertainty in SH biomass burning emission estimates, as can be seen to some extent in Fig. 2. Due to a paucity of Hg emission factor measurements, SH Hg emission estimates are based on Hg emission factor calculations that are dominated by NH data (Friedli et al. 2009; Andreae 2019), which may not be applicable to SH biomes due to differences in vegetation type (Howard et al. 2019) and soil Hg contents (Schneider et al. 2023). While a few emission factor measurements have been made in the SH, methodological differences between studies add considerable uncertainty. In the Amazon, reported emission factors range from 40 to 50 μg Hg (kg fuel)^−1^ to ~ 200 μg Hg (kg fuel)^−1^, with the former based on pre- and post-fire Hg measurement in soil and vegetation pools (Melendez-Perez et al. 2014) and the latter based on the ratio between Hg and carbon monoxide in smoke plumes (and an assumed constant relationship between emission ratios and emission factors; Ebinghaus et al. 2007; Friedli et al. 2009; Koenig et al. 2021). The latter method has been shown to overestimate Hg emissions by up to 60% because emission rates vary with combustion phase and completeness (Howard et al. 2017). Nonetheless, most models have applied these higher Hg emission factors to tropical fires in South America and elsewhere (Friedli et al. 2009; De Simone et al. 2015; Kumar et al. 2018; Shi et al. 2019), potentially overestimating the contribution of fires to the SH atmosphere.

Similarly, recent Hg emission factor measurements from Australian tropical savannas (Desservettaz et al. 2017) and temperate forests (Howard et al. 2019) imply Australian Hg emission factors are much lower than previous estimates (Fisher and Nelson 2020). For Africa, emission factor measurements have only been made in South African savanna and shrubland ecosystems (Brunke et al. 2001; Friedli et al. 2009; Andreae 2019). In equatorial Asia, Hg emission models frequently assume higher Hg emission factors (up to 315 μg Hg (kg fuel)^−1^) than elsewhere in the world (Friedli et al. 2009; Kumar et al. 2018; Shi et al. 2019) due to the prevalence of peatlands, which effectively store Hg (Grigal 2003) and are increasingly vulnerable to fire (van der Werf et al. 2008). However, to the best of our knowledge, Hg emission factors have never been measured for fires (peat or otherwise) in equatorial Asia.

### Deforestation and terrestrial Hg mobilization

Deforestation linked to agriculture, mining, energy infrastructure and urbanization has driven major tree cover loss in the SH + T (Curtis et al. 2018), with estimated deforestation rates since 1990 of 25% in Southeast Asia (Sodhi et al. 2010), 22% in tropical Africa (Aleman et al. 2018), and 11% in the Amazon (ter Steege et al. 2015). Most deforestation occurs in the SH + T (Curtis et al. 2018; Li et al. 2018; FAO 2020; Winkler et al. 2021). The impacts on terrestrial (soil and vegetation) Hg mobilization remain poorly understood, particularly in the SH + T, where they are expected to be significant.

Studies of fire-induced soil Hg mobilization in the SH are limited to Brazil (Lacerda et al. 2004) and Australia (Abraham et al. 2018; Howard et al. 2019). In the Amazon, fire is often used for agricultural purposes as it brings nutrients to otherwise poor soils. This initial fertility, however, is short-lived as the nutrients are easily lixiviated from the soils via rainfall. In the Amazon, it has been shown to take ~ 30 years of fallow for both nutrients (nitrogen, phosphorous) and Hg to return to their pre-deforestation levels in soils once they have been removed via soil erosion and slash-and-burn activities (Farella et al. 2006). Measurements comparing natural and deforested areas in an Amazonian catchment showed deforestation led to a threefold increase in stream Hg output (from 2.9 to 9.3 µg m^−2^) coupled with a decrease of similar magnitude in the soil Hg burden (Fostier et al. 2000). Similarly, Hg in Amazonian soils was found to be nearly two times lower in pasture (34 ng g^−1^) than in forested soils (61.9 ng g^−1^) (Lacerda et al. 2004). In Australia, measurements of Hg concentration in soil before and after a prescribed fire were conducted in a legacy mine site, measured just after the fire and at the end of each season over a one-year period that included an intense rainfall event. The results showed clear soil Hg emission during the fire but also suggested strong lixiviation and runoff effects on Hg mobilization after burning (Abraham et al. 2018). Collectively, these results imply strong Hg re-mobilization after deforestation.

In addition to the lixiviation and erosion processes that release soil Hg to water bodies, deforestation can also increase Hg emission from soils to the atmosphere (Magarelli and Fostier 2005; Almeida et al. 2009; Carpi et al. 2014). A comparison between sites in the NH (New York) and SH (Brazilian Amazon) showed that after forest clearing by burning, gaseous Hg emissions were a factor of two higher in the Amazon (21.2 ± 0.35 ng m^−2^ h^−1^) than in the NH temperate forest (9.13 ± 2.08 ng m^−2^ h^−1^), a difference attributed primarily to stronger light intensity in the tropics (Carpi et al. 2014). Elevated soil emissions have been shown to persist for several months following deforestation and, averaged over an annual timescale, may augment the Hg emissions directly released during Amazonian deforestation fires by an additional 50% (Carpi et al. 2014).

**Fig. 3:**
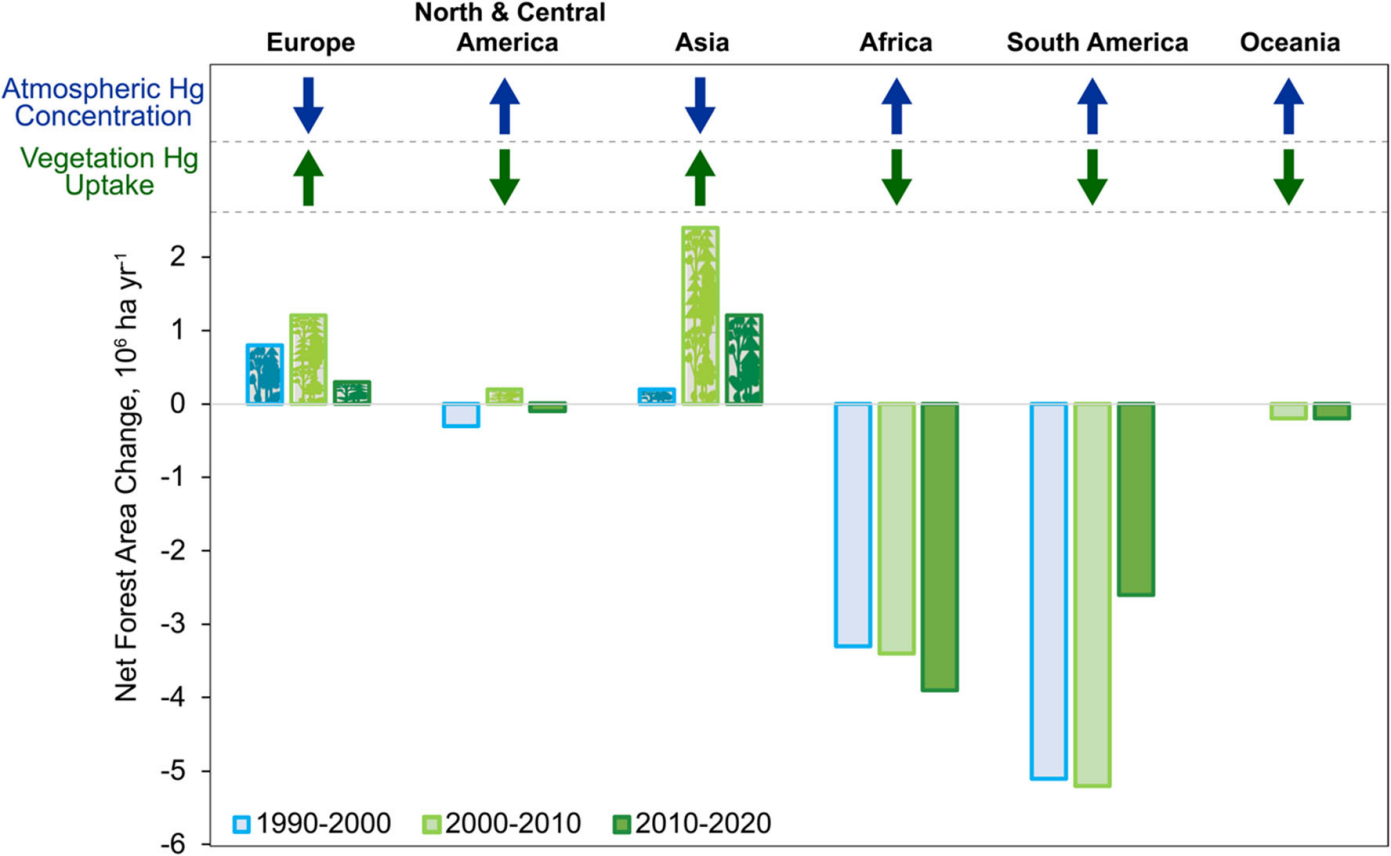
Annual change in net forest area by region and decade (bars), along with conceptual diagram showing the expected implications for vegetation Hg uptake (green arrows) and atmospheric Hg concentrations (blue arrows). Net forest area change is calculated as the sum of all forest losses (deforestation) and all forest gains (forest expansion) in a given period and comes from the FAO Global Forest Resources Assessment 2020 (FAO 2020)

Deforestation also significantly impacts Hg cycling through terrestrial vegetation in the SH. Given the important role of tropical vegetation in removing Hg from the atmosphere (Schneider et al. 2023) and the predominance of deforestation occurring in the SH + T compared to afforestation in the NH (Winkler et al. 2021), the trends in Hg^0^ vegetation uptake are different in the SH than in the NH. In the NH, increasing vegetation cover since 1990 (Nemani et al. 2003; Zhao and Running 2010; Shi et al. 2019; FAO 2020) has strengthened the role of vegetation as a sink for NH atmospheric Hg, removing an estimated additional 140 Mg y^−1^ of Hg^0^ from the atmosphere (Jiskra et al. 2018). Meanwhile, the simultaneous deforestation in the SH is expected to have substantially reduced the atmospheric Hg sink to vegetation, although the magnitude of the effect to date has not previously been quantified. Figure 3 shows annual deforestation/afforestation rates for each region (FAO 2020) along with the anticipated direction of change in the Hg vegetation sink and atmospheric Hg concentrations. The largest changes have occurred in the SH + T, where significant deforestation in African and South America is expected to have decreased the vegetation sink resulting in increased atmospheric concentrations.

The reduction in the vegetation sink is expected to be particularly large in the southern Amazon, where conversion of forests to agriculture is occurring at one of the highest rates in the world (Salazar et al. 2007; FAO 2020; Feinberg et al. 2022). Specifically for the Brazilian Amazon, it has been estimated that about 450,000 km^2^ of forest have been deforested since 1990, with deforestation rates that increased by a further 47–75% between 2019 and 2021, relative to 2018 values (Instituto Nacional de Pesquisas Espaciais 2022). Assuming an average Hg flux of 49 µg m^−2^ y^−1^ reported for Amazonian litterfall (Fostier et al. 2015), we estimate that 22 Mg Hg y^−1^ is no longer being removed from the atmosphere as a result of deforestation in the Amazon to date. As discussed above, the reduced vegetation sink will likely be accompanied by increased soil Hg emissions, further increasing atmospheric Hg concentrations. As an extreme example of the potential consequences of the continuous tropical deforestation on re-mobilization of Hg stored in the terrestrial ecosystem, a recent model study simulating complete conversion of Amazonian rainforest to savanna found a 63% decrease in Hg deposition to the Amazon region, equivalent to more than 400 Mg Hg y^−1^ (Feinberg et al. 2022). In response, the model showed an increased Hg deposition flux to the ocean of 283 Mg Hg y^−1^ that was most pronounced in the Eastern Pacific, with implications for aquatic ecosystems and human exposure pathways (Feinberg et al. 2022).

## Artisanal and small-scale gold mining

Mercury amalgamation is the most common method of gold (Au) recovery used by artisanal and small-scale miners worldwide (Keane et al. 2023) and generates process wastes (tailings) highly contaminated with finely dispersed Hg^0^ droplets (Castilhos et al. 2006). In addition, substantial atmospheric Hg emissions occur from roasting the amalgam to recover the gold. Potentially exacerbating the situation, miners often use cyanide to recover residual gold from these contaminated wastes, leaching soluble Hg cyanide complexes that increase the downstream transport of Hg (Gunson and Veiga 2004; Dewi Krisnayanti et al. 2012). Overall, cyanidation tailings are more water-soluble due to complexation with cyanide. However, the stability and bio-availability of these complexes for methylation remains to be demonstrated, as the cyanide can suppress the microbial activity required for methylation. For example, in the Puyango river in southern Ecuador, the concomitant release of Hg and cyanide wastes resulted in complete suppression of bacterial activity, and consequently Hg methylation, as far as 100 km downstream due to the toxicity of cyanide (Guimaraes et al. 2011). Although Hg^0^ can also be methylated under some conditions (Hu et al. 2013), the methylation process for Hg^0^ also requires microbial activity that can be inhibited by cyanide, if present.

Based primarily on data from the Global Mercury Assessment 2018 (GMA2018; AMAP/UNEP 2019) and Keane et al. (2023), it is estimated that about 70% of the global use of Hg in ASGM occurs in the SH + T. The majority of the use is in Central and South America (~ 55%), with Southeast Asia and Africa having relatively similar usage (15–30% each, depending on the estimation method). The countries with the most Hg use in ASGM are Peru (327 t y^−1^) and Indonesia (427 t y^−1^). Additionally, five South and Central American countries each use more than 100 t y^−1^, with all other countries using < 100 t y^−1^ (AMAP/UNEP 2019). The “small-scale gold mining” terminology used for these activities is somewhat of a misnomer as the mining often occurs in regionally dense pockets of activity, uses large and expensive equipment, and employs a substantial portion of the population in many countries. Globally, ASGM accounts for the livelihood of 100 million people (Keane et al. 2023) and is responsible for a fifth of global gold production.

Keane et al. (2023) compared Hg use in ASGM to gold production from ASGM for countries that had completed their ASGM National Action Plans under the Minamata Convention. While there is scatter in the relationship, the data suggest usage of ~ 1.5 g Hg/g Au produced, an excess over what is theoretically needed (Keane et al. 2023). This high ratio differs between countries because of differences in extraction processes, the price and availability of Hg, and availability of other resources. Total SH + T Hg emissions to the atmosphere from ASGM use are around 1660 t y^−1^ (AMAP/UNEP 2019). Dividing ASGM use between the tropics and the rest of the SH is complicated given that many countries span the Tropic of Capricorn, but it can be concluded that the majority of ASGM activity is confined to the low latitudes, including activities in the tropics, the high Andes, and sub-Saharan Africa.

Most gold mining in the regions outside the tropics in the SH are large-scale commercial activities that do not rely on Hg (e.g., in South Africa and Australia). In the NH, with the exception of China which has substantial ASGM, the top gold producing countries (Russia, USA and Canada) have very little ASGM activity. In contrast, much of the gold producing countries in the SH + T have a larger ASGM than non-ASGM component (although large-scale mining is also prevalent in some of these countries, such as Peru and Indonesia). The majority of the ASGM activities are in developing countries, and the majority of the extraction in developed countries does not rely on Hg use. However, even in countries without significant modern-day ASGM activities, there can be a legacy of Hg contamination from historic Hg use, as occurred in Australia, California and the Yukon in the nineteenth century. In Southern Africa, for example, the use of cyanide for large-scale gold extraction began in 1890, and prior to that used Hg (Fivaz 1988).

In many tropical regions, ASGM is the dominant estimated source of atmospheric Hg emissions, accounting, for example, for 70% of Hg emissions in sub-Saharan Africa and 83% in South America (AMAP/UNEP 2019). This can be contrasted to the global average of 38% of Hg emissions from ASGM. As there is no reported ASGM use in Australia and New Zealand, the ASGM inputs to the SH are regionally variable, with much more ASGM-related emissions from the temperate regions of South America than from Southern Africa and Australasia. In terms of gold recovery efficiency, Hg emissions to the environment have been found to be higher in countries with more limited technology, with recovery higher in South America than in Asia and Central America and lowest in Africa (Seccatore et al. 2014). In terms of the ratio of (Hg used):(Au recovered), the opposite trend was generally apparent, being highest in Africa (Seccatore et al. 2014), although there are countries outside of Africa where this is also the case (e.g., Indonesia, Philippines, Bolivia). There have been many studies examining the local and regional impacts of ASGM activities, but the overall impact of the ASGM-related emissions in the SH specifically has not been well documented.

ASGM miners are primarily exposed to Hg^0^ during amalgamation and refining of the Au-Hg amalgam, primarily by heating to vaporize the Hg^0^. Besides this direct exposure and its atmospheric release, Hg release to aquatic systems, either during various stages of the amalgamation process or as a result of washoff during rain and flood events, is an important route for human exposure, primarily to MeHg. Any Hg released to aquatic systems, either as Hg^0^ or as ionic Hg, can be methylated to MeHg, which can bioaccumulate up the food chain resulting in human exposure. Thus, communities downstream of Hg inputs may have a higher exposure through fish consumption because of the elevated MeHg burden. A key factor for human exposure is the suitability of the environment for Hg methylation. For example, in French Guiana, the presence of a reservoir with anoxic bottom waters was found to have exacerbated the transformation to MeHg and increased MeHg concentrations in fish. Such enhanced methylation was more significant than the impact of ASGM-derived Hg inputs to the river (Boudou et al. 2005). In terrestrial ecosystems, forests near ASGM sites have been shown to sequester significant quantities of ASGM-emitted Hg in both vegetation and soil, and this elevated Hg affected terrestrial food webs (Gerson et al. 2022).

One issue that complicates the determination of the impacts from ASGM Hg release to aquatic and terrestrial systems is the fact that Hg concentrations are higher in background soils in remote locations in South America than elsewhere (Schneider et al. 2023). Therefore, the indirect impacts of ASGM, such as deforestation, soil disturbance and soil release to aquatic environments, are potentially additional sources of Hg to rivers and coastal waters. This was initially highlighted in the Amazon (Roulet et al. 1998) but could not be definitively confirmed at the time. More recently, stable Hg isotope signatures have confirmed that, in the Amazon, the Hg input to aquatic systems as a result of soil erosion can be more important than the direct losses of the Hg used in ASGM (Schudel et al. 2018). As the background levels of Hg in soils in South America are relatively high compared to other SH continents (Schneider et al. 2023), the extent to which this is a problem in other parts of the SH + T is not known. Indeed, in many African countries, ASGM also causes deforestation and soil erosion (Ncube-Phiri et al. 2015; Mhangara et al. 2020; Girard et al. 2022), and there is a concomitant production of charcoal for energy needs (Zulu and Richardson 2013), all of which could exacerbate the overall impact of ASGM by increasing Hg releases from associated activities above those from direct use in ASGM. The impact of collateral activities on Hg releases, in addition to the ASGM impact, has not been adequately studied.

**Fig. 4:**
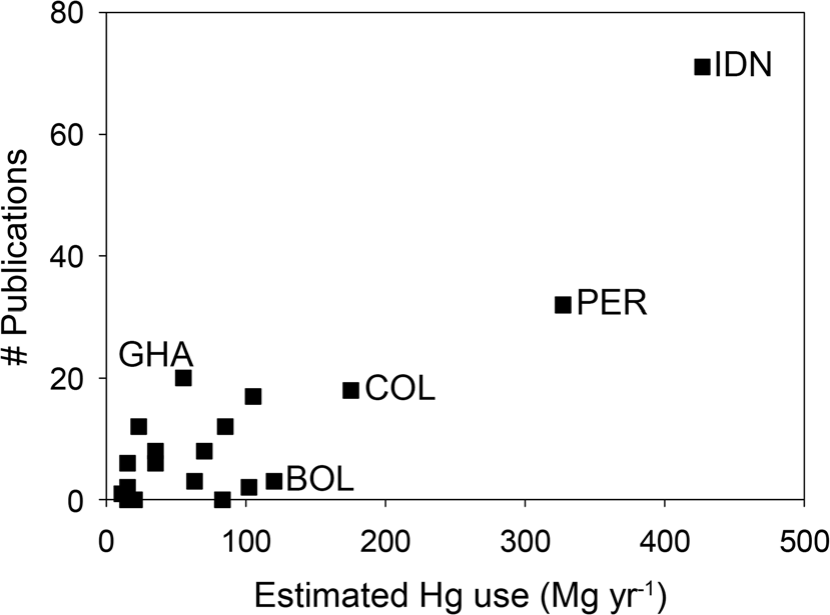
Number of scientific articles on Hg use in ASGM as a function of projected mercury use in ASGM for countries with known ASGM activities. *BOL* Bolivia, *COL* Colombia, *GHA* Ghana, *IDN* Indonesia, *PER* Peru. Data sources: projected Hg use from the Global Mercury Assessment Report 2018 Technical Background Report (AMAP/UNEP 2019) Table A3.2.1; number of scientific articles on ASGM and mercury use in the country from Scopus as of 18 November 2022

In addition, in trying to understand the impacts of ASGM in the SH + T, there is a disconnect between the extent of Hg use in ASGM in a country and the number of scientific studies of the impacts. While limited in scope (capturing only indexed, English-language, peer reviewed articles), a Scopus search with the words “ASGM, mercury and [country]” in the title, abstract, and/or keywords is illustrative of this disconnect. The search yielded the data plotted in Fig. 4 (data provided in the Supplementary Information), which includes 8 South American countries (Bolivia, Brazil, Colombia, Ecuador, Guyana, Peru, Suriname, Venezuela), 10 African countries (Burkina Faso, Democratic Republic of Congo, Ghana, Guinea, Mali, Nigeria, Sierra Leone, Sudan, Tanzania, Zimbabwe), and 3 Asian countries (Indonesia, Myanmar, Philippines). Indonesia has the highest Hg use in the SH + T and has the highest number of studies, followed by Peru. Other South American countries (e.g., Bolivia and Venezuela) have relatively high Hg usage but very few studies were identified with the search keywords. Ghana has the opposite situation, with the relative number of studies greater than the relative Hg usage. Overall, this analysis highlights the discrepancies in the research focus in the SH + T. In many instances, especially in Africa, the focus of an individual research group or scientist is the driver of the extent to which the problem is studied, rather than the expected magnitude of the Hg use in ASGM. Perhaps, with the increase in the implementation of National Action Plans under the Minamata Convention, the distribution of future research studies will more accurately reflect the overall Hg usage.

Globally, the impacts of Hg use in ASGM are well-documented (AMAP/UNEP 2019) and are not elaborated here. The impacts of ASGM in the tropics on the release of Hg to the atmosphere as well as to rivers, lakes and coastal waters are a clear concern for local and regional human exposure to MeHg. Conditions in the tropics likely favor more net Hg methylation than in temperate regions (Mason 2012). More studies are needed to compare the exposure of those directly involved in ASGM activities to those who may be harvesting fish and other aquatic resources downstream of such activities. Differences in diet are also important in terms of exposure from sources other than fish, such as rice, and should be considered.

Overall, the direct impact of ASGM on Hg exposure is not globally distributed but is more focused in tropical countries in both hemispheres. Factors increasing the potential exposure from ASGM are: (1) the levels of gold and Hg in soils/sediments in these countries; (2) the lack of employment in many rural areas that results low-tech employment, such as in the ASGM industry; (3) the low cost and lack of governmental control of Hg import and ASGM activity; (4) the high relative price for gold; and (5) the relatively low point source inputs of Hg in ASGM areas, such as Hg from coal burning for electricity generation. As noted above, reservoirs constructed for electricity generation can enhance the conversion of Hg into MeHg. While there continues to be widespread Hg use in ASGM, there is some indication that there is a shift to using alternative technologies and extraction techniques. However, one increasingly widespread approach, the use of cyanide for gold extraction, reduces one environmental problem but creates another as discussed above (Tulasi et al. 2021). There is an urgent need to develop gold extraction methods that are more environmentally friendly and that have much lower impacts on human health, as discussed in detail in Keane et al. (2023).

## Industrial sources

**Fig. 5:**
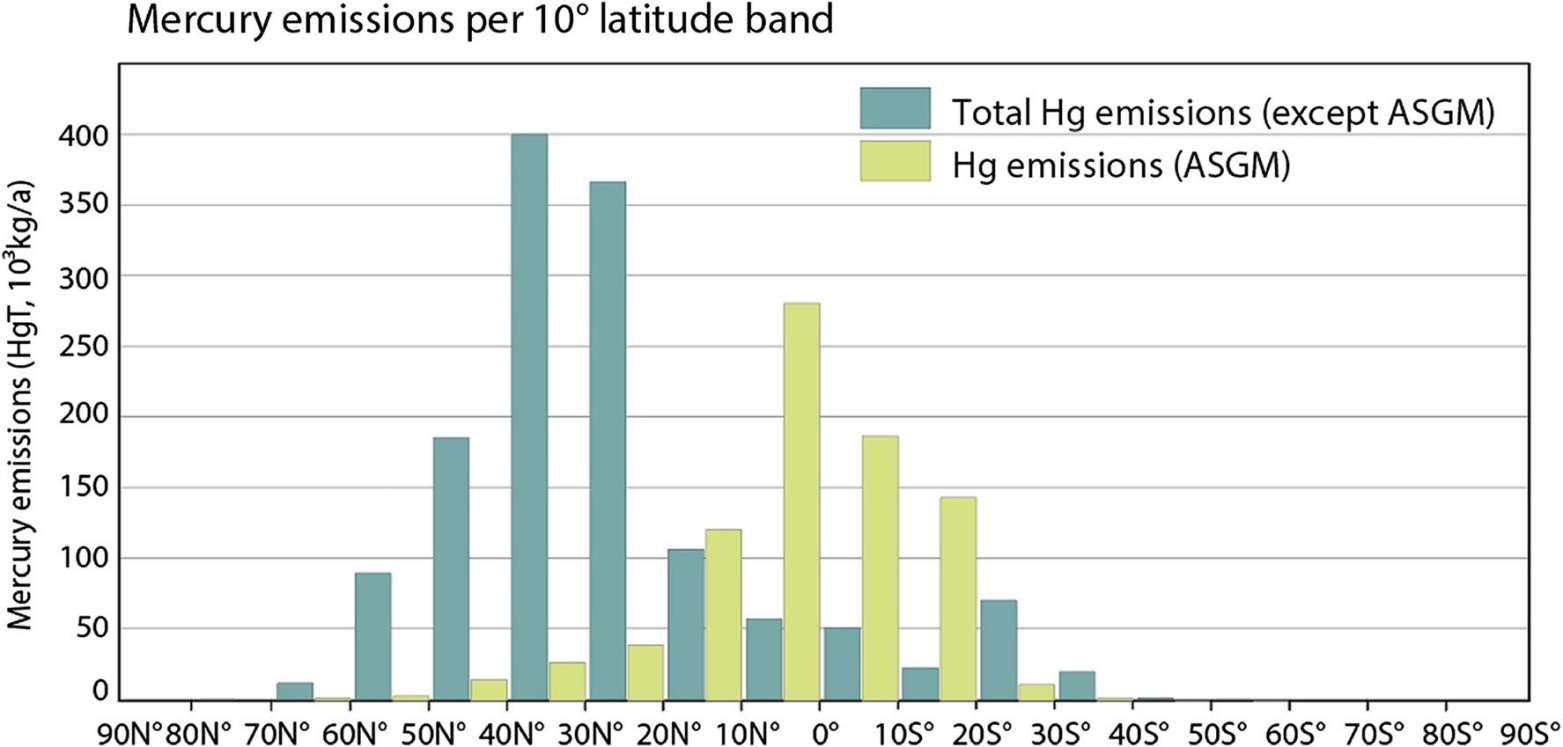
Anthropogenic Hg emissions by latitude, separated into ASGM (light green) and non-ASGM (dark green) emissions. Reprinted from Atmospheric Environment, Vol 211, Steenhuisen and Wilson, Copyright (2019), with permission from Elsevier

With only 10% of the global population located in the SH, non-ASGM anthropogenic Hg emissions are much lower in the SH than in the NH. Within the SH, these non-ASGM emissions are also much lower than ASGM emissions (Steenhuisen and Wilson 2019), as shown in Fig. 5. Nonetheless, Hg pollution from industrial sources can be locally significant, particularly in the more populated regions of the SH (Fostier and Michelazzo 2006; Higueras et al. 2014; Belelie et al. 2019; Schofield et al. 2021), likely exacerbated by less stringent Hg regulatory policies as well as less advanced emissions abatement technologies.

The largest sources of industrial Hg emissions in the SH are coal combustion and non-ferrous metals production, with regional contributions from legacy Hg contamination from historical mining and Hg processing facilities (Walters et al. 2011) and municipal waste and e-waste dumps and processing facilities (Nipen et al. 2022). While industrial emissions have declined across much of the NH, driving decreasing trends in observed atmospheric Hg in North America and Europe (Zhang et al. 2016), SH industrial emissions have continued increasing in recent years (AMAP/UNEP 2019). These projections have not been corroborated empirically, as SH air measurement records are either not sufficiently long-term to quantify trends or are more sensitive to natural emissions than anthropogenic emissions (e.g., Martin et al. 2017). Here we focus on the two industrial Hg sources estimated to have the largest emissions in the SH: coal-fired power stations and non-ferrous metal production.

### Coal-fired power stations

The amount of Hg emitted by coal-fired power stations depends on coal consumption, the Hg contents of incinerated coals, and the fraction of Hg captured in the emission abatement technologies installed at power stations. These factors vary widely across SH countries. Nearly 90% of SH coal is consumed by (in decreasing order) South Africa, Indonesia, and Australia (bp 2022). Coal consumption in these countries is significant at both hemispheric and global scales, with South Africa ranked as the 5th largest consumer of coal in the world (following China, India, the United States, and Japan), and Indonesia and Australia ranked 7th and 11th, respectively (bp 2022). All three countries are also major coal exporters (bp 2022), potentially contributing to Hg emissions in other parts of the world.

Table 1 shows the measured coal Hg content for these three countries (with other countries in the SH + T included for reference). In addition to having the highest coal consumption in the SH, the Hg content in South African coals is higher than the global average (Lusilao-Makiese et al. 2012; Garnham and Langerman 2016; Mathebula et al. 2020) and reportedly higher than any other country except India (AMAP/UNEP 2019). Emission abatement technologies used in South African power stations include electrostatic precipitators (ESPs) and fabric filters, which reduce Hg emissions by about 25–35% and 50–90%, respectively (Garnham and Langerman 2016). These technologies are less efficient than wet flue gas desulfurization (wetFGD) technologies used in many industrialized NH countries (Malmgren and Riley 2012; Singhal 2018; van Ewijk and McDowall 2020). Overall, Hg emissions from South African coal-fired power plants are estimated at 17–40 Mg y^−1^ (Garnham and Langerman 2016; AMAP/UNEP 2019), making South Africa one of the largest sources of Hg emissions from coal-fired power stations in the world and almost certainly the largest source in the SH (AMAP/UNEP 2019). South African power stations are being retrofitted with improved emission abatement technologies; however, future Hg emissions are projected to decline by only 6–13% (Garnham and Langerman 2016).

**Table 1:** Hg contents in SH coals

Location	Coal type	Mean ± standard deviation (mg kg^−1^)	Range (mg kg^−1^)	References
Major coal consuming countries				
South Africa	Bituminous	0.15 ± 0.05	0.010–0.49	Mathebula et al. (2020)
	Bituminous	0.27 ± 0.11	0.12–0.70	Garnham and Langerman (2016)
	Bituminous	0.20 ± 0.03	0.14–0.30	Lusilao-Makiese et al. (2012)
	Unknown	0.16 ± 0.17	0.023–0.83	Tewalt et al. (2010)
Australia	Bituminous	0.04	0.01–0.13	Dale (2003); Riley et al. (2005); Nelson (2007)
	Brown	0.08 (dry) = 0.032 (moisture corrected)	Not available	Brockway and Higgins (1991), Nelson (2007)
	Bituminous	0.065 ± 0.10	0.011–0.31	Tewalt et al. (2010)
	Bituminous	0.043 ± 0.003	0.040–0.047	Schneider et al. (2021)
	Lignite	0.071	One sample	Schneider et al. (2021)
Indonesia	Unknown	0.056	0.011–0.23	Basel Convention Regional Centre for South East Asia and Stockholm Convention Regional Centre Indonesia (2017)
	Unknown	0.10 ± 0.062	0.022–0.19	Tewalt et al. (2010)
Other SH + T countries				
South America				
Argentina	Bituminous	0.20 ± 0.34	0.021–0.96	Tewalt et al. (2010)
Bolivia	Unknown	0.05	One sample	Tewalt et al. (2010)
Brazil	Bituminous	0.19 ± 0.15	0.040–0.44	Tewalt et al. (2010)
	Subbituminous	0.30 ± 0.23	0.064–0.94	Tewalt et al. (2010)
Chile	Bituminous	0.069 ± 0.058*	< 0.03–2.2	Tewalt et al. (2010)
	Subbituminous	0.033 ± 0.017	0.022–0.057	Tewalt et al. (2010)
Chile	Unknown	0.23 ± 0.55	0.022–2.2	Tewalt et al. (2010)
Colombia	Bituminous	0.015 ± 0.00*	< 0.03	Tewalt et al. (2010)
	Subbituminous	0.041 ± 0.037*	< 0.03–0.067	Tewalt et al. (2010)
	Unknown	0.064 ± 0.045*	< 0.03–0.17	Tewalt et al. (2010)
Peru	Anthracite	0.25 ± 0.19	0.041–0.63	Tewalt et al. (2010)
Venezuela	Unknown	0.20 ± 0.51*	< 0.03–2.1	Tewalt et al. (2010)
Sub-Saharan Africa				
Botswana	Unknown	0.10 ± 0.027	0.041–0.15	Tewalt et al. (2010)
Nigeria	Bituminous	0.11 ± 0.067	0.036–0.24	Tewalt et al. (2010)
	Subbituminous	0.033 ± 0.022	0.013–0.085	Tewalt et al. (2010)
Tanzania	Unknown	0.12 ± 0.059	0.02–0.22	Tewalt et al. (2010)
	Subbituminous	0.10 ± 0.053	0.041–0.16	Tewalt et al. (2010)
Zambia	Unknown	0.52 ± 0.92*^+^	< 0.03–3.6	Tewalt et al. (2010)
Zimbabwe	Unknown	0.068 ± 0.046*	< 0.03–0.15	Tewalt et al. (2010)
Oceania				
New Zealand	Bituminous	0.050 ± 0.037*	< 0.03–0.10	Tewalt et al. (2010)
	Subbituminous	0.076 ± 0.025	0.058–0.094	Tewalt et al. (2010)
	Lignite	0.076 ± 0.029	0.021–0.13	Tewalt et al. (2010)

For reference, AMAP/UNEP (2019) reports a generic default value (indicative of a global average) of 0.15 mg kg^−1^ and NH country-specific values of 0.05 (Japan), 0.05–0.08 (Republic of Korea), 0.07 (Canada), 0.06–0.1 (Russian Federation), 0.1 (USA), 0.17–0.19 (China), and 0.14–0.29 (India) mg kg^−1^

^*^Dataset included values of “< 0.03”. When calculating averages and standard deviations, it was assumed that 0.03 was the detection limit, and these values were set to 0.015 (half the detection limit)

^+^Includes one outlier at 3.6 mg kg^−1^. Excluding the outlier would give 0.28 ± 0.24 (range < 0.03–0.81)

In contrast, the coals of Australia and Indonesia have natural Hg loads that are much lower than the global average (Nelson 2007; Basel Convention Regional Centre for South East Asia and Stockholm Convention Regional Centre Indonesia 2017) as shown in Table 1. In Australia, coal-fired power plants still account for nearly 40% of domestic anthropogenic Hg emissions, despite emission declines following the recent closure of several major power plants (MacFarlane et al. 2022). Although Hg emissions from Australian power plants are small on a global scale (~ 3.5 Mg yr^−1^), the per capita emissions are larger than in similarly industrialized countries in the NH (AMAP/UNEP 2019; MacFarlane et al. 2022). Australia relies on a mix of black and brown coal, with emission abatement technologies (fabric filters and ESPs) reducing the Hg emissions by only 20% for black coals and 2% for brown coals (Nelson 2007; Department of Sustainability, Environment, Water, Population and Communities 2012; Nelson et al. 2012; Fisher and Nelson 2020; MacFarlane et al. 2022). In contrast, power stations in similarly industrialized countries in the NH (e.g., the US) typically use more efficient wetFGD technologies (Schneider and Sinclair 2019). However, it is worth noting that the capture efficiencies assumed in Australia are based on U.S. data and may not accurately reflect the capture performance in other locations. Furthermore, the main determinant of Hg capture is the amount of unburnt carbon in the flue gas, and so lower efficiency coal combustion may result in higher Hg capture efficiency via increased partitioning to easier-to-capture reactive Hg (U.S. Environmental Protection Agency 2005).

In Indonesia, there are no regulatory standards for Hg (and emission regulations for other pollutants are limited; Basel Convention Regional Centre for South East Asia and Stockholm Convention Regional Centre Indonesia 2017). Most power stations in Indonesia use either ESPs or a combination of ESP and FGD, which capture 3.5–27% and 55% of the Hg emitted, respectively (Basel Convention Regional Centre for South East Asia and Stockholm Convention Regional Centre Indonesia 2017). Hg emissions from power stations in Indonesia total ~ 3–4 Mg y^−1^, on par with power station Hg emissions from Australia, despite Indonesia being a much more populous country. It is projected that Hg emissions in Indonesia will grow until 2024, after which reductions in coal use to combat climate change are expected to have a co-benefit for Hg emission (Basel Convention Regional Centre for South East Asia and Stockholm Convention Regional Centre Indonesia 2017).

### Non-ferrous metals production

Industrial-scale production of non-ferrous metals releases Hg to the environment during both the mining and post-mining processing stages. Unlike for ASGM, the industrial mining process itself is not considered to be a significant source of Hg emissions to the atmosphere; however, it can be an important source of Hg releases to water and soil via oxidation and subsequent leaching of exposed waste rock and tailings created during mining (UNEP Global Mercury Partnership 2022). Subsequent thermal processing (e.g., smelting and roasting) releases Hg to the atmosphere and to land and water. For many non-ferrous metals (excluding gold), mined ore is converted to concentrate and transported to a different facility for processing. This leads to an important observation that most Hg emissions from non-ferrous metals production do not occur at the source of mining, but rather at the processing facility – which may be in a completely different country (UNEP Global Mercury Partnership 2022).

The extent to which the disjoint between ore mining and concentrate processing affects emission estimates for countries in the SH is poorly understood. However, based on current estimates compiled for the GMA2018, air emissions from non-ferrous metal production in SH countries are globally significant, with roughly half of total global industrial (non-ASGM) gold production and copper production emissions attributed to countries in South America, sub-Saharan Africa, and Oceania (mainly Indonesia).

**Fig. 6:**
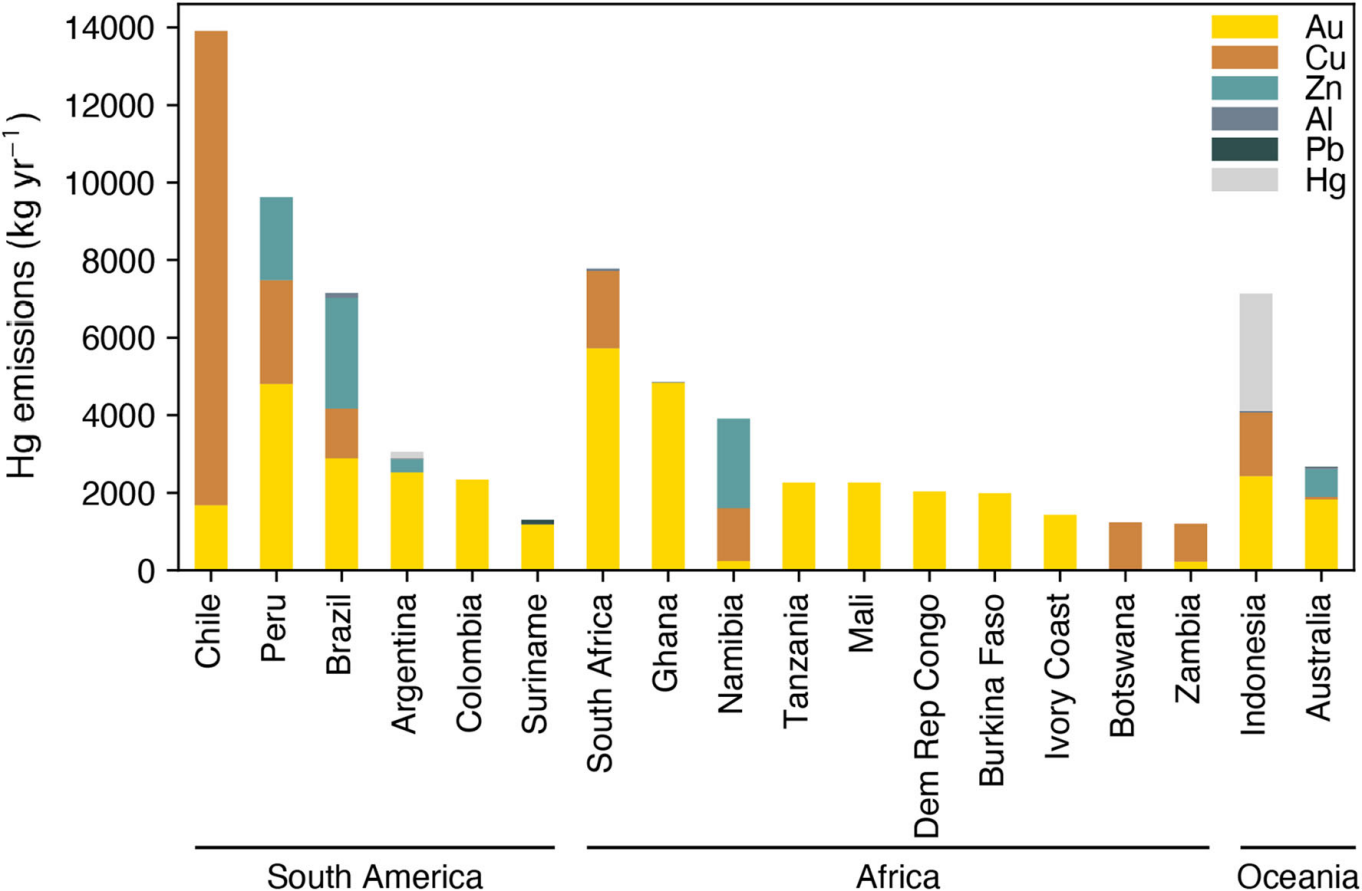
Mercury emissions from non-ferrous metal production in South America, Africa, and Oceania, colored by metal produced. For clarity, only countries with estimated emissions of more than 1 Mg y^−1^ are shown. Data source: GMA2018 Technical Background Report (AMAP/UNEP 2019)

Hg emissions and releases from the non-ferrous metals sector are a particular concern in South America. Chile and Peru contain large copper deposits (more than a quarter of the known global reserves), and large amounts of lead and zinc are found in Peru, Brazil, Bolivia, and Argentina (UNEP Global Mercury Partnership 2022). Estimates compiled for the GMA2018 suggest that South American non-ferrous metal production is responsible for nearly 40 Mg y^−1^ of Hg emissions to the atmosphere (Fig. 6), accounting for more than half of total non-ASGM emissions from South America (AMAP/UNEP^ 2019^). Roughly equal amounts come from production of non-ASGM gold (16.7 Mg y^−1^) and copper (16.2 Mg y^−1^) followed by zinc (5.3 Mg y^−1^), with small contributions from lead and aluminum. As expected from the geographic distribution of the ore reserves, the majority of emissions come from Chile (14 Mg y^−1^), followed by Peru (10 Mg y^−1^) and Brazil (7 Mg y^−1^). For many South American countries (Bolivia, Brazil, Colombia, Guyana, Peru, and Suriname), non-ferrous metal production is the largest non-ASGM emission source. In Argentina and Chile, ASGM does not occur, and so non-ferrous metal production is the dominant source overall (AMAP/UNEP 2019).

In sub-Saharan Africa, non-ferrous metal production emissions are 34 Mg y^−1^ (Fig. 6), accounting for roughly a third of non-ASGM emissions in the region (AMAP/UNEP 2019). Here, industrial gold production emissions are dominant (25.5 Mg y^−1^), followed by production of copper (5.5 Mg y^−1^), zinc (2.3 Mg y^−1^), and aluminum (< 1 Mg y^−1^). The biggest contributions come from South Africa (8 Mg y^−1^), Ghana (5 Mg y^−1^), and Namibia (4 Mg y^−1^). Current estimates suggest that non-ferrous metal production is the largest non-ASGM source of Hg emissions for Burkina Faso, the Democratic Republic of Congo, Eritrea, Ghana, Guinea, Liberia, Mali, Tanzania, and Zimbabwe. It is the dominant source overall (larger than ASGM emission) in Botswana, Ivory Coast, Mauritania, Namibia, and Zambia (AMAP/UNEP 2019).

Non-ferrous metal production is also a significant source of Hg releases to aquatic systems. While country-level estimates are not available for aquatic releases, regional estimates from the GMA2018 suggest non-ferrous metal production accounts for roughly a third of non-ASGM Hg releases in Sub-Saharan Africa (mainly from industrial-scale gold production) and just under half in South America (a mix of gold and other metals production). Globally, 40% of total non-ASGM releases to water (13% of total releases including ASGM) are attributed to non-ferrous metals production according to the GMA2018 (AMAP/UNEP 2019). Releases to land and soil may be even larger; for example, industrial-scale gold mining is thought to release 45 times more Hg to soil than to water (AMAP/UNEP 2019). These pathways remain highly uncertain, and the methodology employed by the GMA2018 may in fact overestimate releases to land and water (UNEP Global Mercury Partnership 2022). The GMA2018 employs a mass balance approach, in which all Hg in the ore/concentrate that is not emitted to air is assumed to be released to the environment. In reality, much of this Hg may be captured in pollution control devices and managed by the facility in a way that prevents release to the environment (e.g., secure storage as cinnabar in pupose-built facilities; UNEP Global Mercury Partnership 2022). Bottom-up release estimates based on facility reporting are much lower (UNEP Global Mercury Partnership 2022), but these are not generally peer-reviewed or made publicly available. Differing environmental standards, monitoring, and enforcement likely add significant country-level variability to the fraction of input Hg ultimately released to land and water.

These pathways remain highly uncertain and poorly quantified (UNEP Global Mercury Partnership 2022). Given the relative importance of SH countries for global gold and copper production and for Hg emission to air, these countries are expected to provide a globally significant contribution to Hg releases to land and water as well.

Significant growth in the non-ferrous sector is expected over the next 30 years, with the demand for metals doubling or tripling by 2050 (Elshkaki et al. 2018). With large metal ore reserves and existing processing facilities, it is highly likely that SH countries will continue to provide a significant share of the metals required to meet this need. Without the implementation of new Hg mitigation measures, the non-ferrous sector could become an increasingly important source of SH Hg pollution in air, land, and water (UNEP Global Mercury Partnership 2022).

## Conclusion and future research needs

In this paper, we have reviewed current understanding of the ways in which human activities have perturbed the sources and cycling of Hg in the SH and tropics (SH + T), with particular focus on differences relative to the NH. Historically, sources of legacy Hg emissions such as Hg production sites and industrial-scale end-uses have been more prevalent in the NH than in the SH + T, but there is limited data at present on the magnitude of this imbalance. Fire and deforestation are prevalent in the tropics, mobilizing Hg stored in the terrestrial biosphere via direct emission to air, leaching and erosion releases to water bodies, and removal of vegetation that would otherwise serve as a sink for atmospheric Hg. ASGM continues to be the dominant source of Hg inputs to the environment in many tropical countries, with subsequent exposure routes dependent on regional differences in natural soil Hg contents and water bodies’ methylation potential. Coal-fired power stations continue to be a major Hg source in South Africa, Australia, and Indonesia, where substantial coal use is coupled with less advanced abatement technologies than used in NH industrialized countries. While the power station source is declining, industrial production of non-ferrous metals is a large and growing contributor to Hg emission and releases across the SH.

The unequal distribution of Hg research continues to hamper our understanding of Hg sources, cycling and the response to anthropogenic perturbations in the SH + T. The very large uncertainties in all processes discussed here (with perhaps the exception of coal-fired power station emissions) make it difficult to reliably quantify SH-specific Hg budgets, which impedes our ability to model present-day Hg distributions. Current Hg models are unable to reproduce observed atmospheric Hg concentrations at most land-based SH sites (Zhang and Zhang 2022), which themselves are far from a representative sample of the diversity of SH environments (Schneider et al. 2023). As a result, critical gaps remain that hinder informed policy-making efforts to support implementation of the Minamata Convention.

This synthesis has identified a number of key gaps that require future research prioritization. In the case of legacy Hg emissions, it is necessary to expand the base of historical data to progress beyond the limitations of a silver/gold historical scenario. Archival research into the history of Hg production losses, global trade and markets will provide more detailed, country-specific accounting of legacy Hg sources in both hemispheres, and thus of their comparative and distinct impacts on the environment. More SH Hg deposition studies are needed to address biases caused by the small number of sites and reliance on peat core measurements, with a particular need to sample lakes that are not directly downwind of major preindustrial Hg emission sources and to sample inland areas of the SH that are less affected by oceanic emissions.

For fires and deforestation, current estimates of atmospheric Hg emission from SH fires rely heavily on emission factor measurements made in the NH and (in a few cases) the Amazon and Australia. Similarly, measurements of post-fire Hg release to water bodies have been restricted to Brazil. Measurements of fire and deforestation Hg impacts are urgently needed for other regions and ecosystems, particularly peatland fires in equatorial Asia, tropical forest fires in Africa, and temperate fires in southern South America. In addition, modelling studies to date have largely focused on quantifying the direct emissions of Hg to the atmosphere during fires. Longer-term impacts of fire and deforestation, including enhanced gaseous Hg emission from soil and reduction in atmospheric Hg uptake by vegetation, have received less attention. Improved understanding and model parameterization of these processes should be prioritized for accurate prediction of SH mercury cycling and its response to change.

For ASGM, while the use of Hg occurs primarily in tropical countries, it remains a major global source of Hg to the atmosphere that impacts all countries. There is a need for future research to examine and demonstrate the consequences of Hg use in ASGM and the associated environmental impacts of activities related to ASGM use. Such an effort requires global action given that current studies are not directly related to use but are influenced by other factors. The development of inexpensive alternatives to Hg are needed in conjunction with a global effort to convince miners to change to a less toxic and less environmentally damaging methodology. The associated impacts of deforestation and soil erosion will remain, and the long-term impacts need further study. This is a concern mostly for heavily forested countries and those with higher natural levels of Hg in soils, such as in South America. Finally, while site-specific studies allow for demonstration of impact in a particular location and highlight the large-scale impacts of Hg use, there is a need for a global perspective and action through the Minamata Convention and other international organizations to dissuade the use of Hg in ASGM.

For industrial emissions, while coal-fired power stations are currently a major Hg emission source in some countries, future emission declines are anticipated as power stations either upgrade technologically or close in response to the overlapping concerns of climate change, air pollution, and Hg pollution. The resultant trends in Hg emissions remain uncertain, and there will be a future need to re-evaluate power station emissions in light of these changes. Non-ferrous metal production is an important and growing source in many SH countries, but there are large uncertainties on emissions and releases. Ore Hg content can vary significantly between locations, and Hg measurements in ores, air emissions, and waste materials at facilities across the SH are needed to constrain this source.

Finally, an ongoing challenge for Hg science and policy in the SH remains the lack of long-term, continuous measurements. Understanding and predicting the response to change—including further deforestation, climate change impacts, and actions to support the Minamata Convention—will require measurement records in diverse SH environments that allow us to track changes over time. Despite being highlighted as a priority in the last synthesis effort (Obrist et al. 2018; Selin et al. 2018), such measurements remain extremely limited. Establishing and supporting ongoing Hg measurement networks across the SH should be a priority for both the scientific community and governments committed to evaluating the effectiveness of the Minamata Convention.

## Supplementary Information

Below is the link to the electronic supplementary material.

Supplementary file1 (PDF 81 kb)

Supplementary file1 (XLSX 128 kb)
