# Supplementary material for: A synthesis of mercury research in the Southern Hemisphere, part 2: Anthropogenic perturbations
Source: Ambio. 2023 Mar 23;52(5):918–37. doi: 10.1007/s13280-023-01840-5 (PMC10073395; doi:10.1007/s13280-023-01840-5)
Supplement: Supplementary file 1 — Supplementary file1 (PDF 81 kb) [file 13280_2023_1840_MOESM1_ESM.pdf]

Ambio

Electronic Supplementary Material

Title: **A Synthesis of Mercury Research in the Southern Hemisphere, Part 2: Anthropogenic Perturbations**

Authors: Jenny A. Fisher, Larissa Schneider, Anne-Hélène Fostier, Saul Guerrero, Jean Remy Davée Guimarães, Casper Labuschagne, Joy J. Leaner, Lynwill G. Martin, Robert P. Mason, Vernon Somerset, Chavon Walters

Table S1. Underlying data sources for fire emission estimates shown in Figure 2. For more details of other parameters (e.g., land cover maps, fuel loads, combustion completeness), see the original references.

Figure 2 Label	Reference	Fire Activity Source	Emission Factors (EFs)	Notes
Friedli	Friedli et al., 2009	From GFEDv2 (based on MODIS burned area product)	Five EFs (based where possible on measurement compilations): boreal forest, temperate forest, tropical forest, tropical peat forest, nonforest	For details of the measurements on which EFs are based, see Friedli et al. 2009, Supporting Information
De Simone 1 (FINN)	De Simone et al., 2015	From FINNv1.0 (based on MODIS active fire product)	One EF used globally (based on global average EF from Friedli et al., 2009)	More detailed EFs were available but only used in sensitivity studies
De Simone 2 (GFAS)	De Simone et al., 2015	From GFASv1.0 (based on MODIS fire radiative power product)	Same as De Simone 1	
De Simone 3 (GFED)	De Simone et al., 2015	From GFEDv3.1 (based on MODIS burned area product)	Same as De Simone 1	
Kumar	Kumar et al., 2018	From a statistical model of burned area based on modelled fire frequency from Huang et al., 2015	Based on Friedli et al., 2009 but with downward revision for tropical forests based on Melendez-Perez et al., 2014	While the statistical model is trained on MODIS active fire counts and burned area from GFEDv4, the final burned area estimate differs substantially from GFEDv3, GFEDv4, and FINN (by 50% to more than 200%, depending on the region). For more details, see Kumar et al., 2018, Table 1a

Shi	Shi et al., 2019	From MODIS burned area product, combined with MODIS active fire product for cropland only	Three EFs (tropical region only): forest, shrubland, and grassland + agricultural waste	EFs are based on averages from multiple studies including Kumar et al., 2018, Friedli et al., 2009, and others. For more details, see Shi et al., 2019, Table 1. Note that values used from Kumar et al. were treated as emission factors (µg Hg/kg fuel in Shi t al. 2019 Table 1) whereas the original source states these as annual Hg emissions (Mg Hg/yr in Kumar et al. Table 3)

References

De Simone, F., S. Cinnirella, C. N. Gencarelli, X. Yang, I. M. Hedgecock, and N. Pirrone. 2015. Model study of global mercury deposition from biomass burning. Environmental Science & Technology 49: 6712–6721. doi:10.1021/acs.est.5b00969.

Friedli, H. R., A. F. Arellano, S. Cinnirella, and N. Pirrone. 2009. Initial estimates of mercury emissions to the atmosphere from global biomass burning. Environmental Science & Technology 43: 3507–3513. doi:10.1021/es802703g.

Huang, Y., S. Wu, and J. O. Kaplan. 2015. Sensitivity of global wildfire occurrences to various factors in the context of global change. Atmospheric Environment 121: 86–92. doi:10.1016/j.atmosenv.2015.06.002.

Kumar, A., S. Wu, Y. Huang, H. Liao, and J. O. Kaplan. 2018. Mercury from wildfires_ Global emission inventories and sensitivity to 2000–2050 global change. Atmospheric Environment 173: 6–15. doi:10.1016/j.atmosenv.2017.10.061.

Melendez-Perez, J. J., A. H. Fostier, J. A. Carvalho, C. C. Windmöller, J. C. Santos, and A. Carpi. 2014. Soil and biomass mercury emissions during a prescribed fire in the Amazonian rain forest. Atmospheric Environment 96: 415–422. doi:10.1016/j.atmosenv.2014.06.032.

Shi, Y., A. Zhao, T. Matsunaga, Y. Yamaguchi, S. Zang, Z. Li, T. Yu, and X. Gu. 2019. High-resolution inventory of mercury emissions from biomass burning in tropical continents during 2001–2017. Science of The Total Environment 653: 638–648. doi:10.1016/j.scitotenv.2018.10.420.
